# Two New Genera of Planktonic Ciliates and Insights into the Evolution of the Family Strombidiidae (Protista, Ciliophora, Oligotrichia)

**DOI:** 10.1371/journal.pone.0131726

**Published:** 2015-06-29

**Authors:** Weiwei Liu, Zhenzhen Yi, Dapeng Xu, John C. Clamp, Jiqiu Li, Xiaofeng Lin, Weibo Song

**Affiliations:** 1 Laboratory of Protozoology, Key Laboratory of Ecology and Environmental Science in Guangdong Higher Education, South China Normal University, Guangzhou, China; 2 Laboratory of Protozoology, Institute of Evolution and Marine Biodiversity, Ocean University of China, Qingdao, China; 3 Key Laboratory of Tropical Marine Bio-resources and Ecology, South China Sea Institute of Oceanology, Chinese Academy of Science, Guangzhou, China; 4 State Key Laboratory of Marine Environmental Science, Institute of Marine Microbes and Ecosphere, Xiamen University, Xiamen, China; 5 Department of Biology, North Carolina Central University, Durham, United States of America; Queensland University of Technology, AUSTRALIA

## Abstract

Oligotrich ciliates are common marine microplankters, but their biodiversity and evolutionary relationships have not been well-documented. Morphological descriptions and small subunit rRNA gene sequences of two new species representing two new strombidiid genera, *Sinistrostrombidium cupiformum* gen. nov., sp. nov. and *Antestrombidium agathae* gen. nov., sp. nov. are presented, and their taxonomy and molecular phylogeny are analyzed. *Sinistrostrombidium* gen. nov. is characterized by a sinistrally spiraled girdle kinety and a longitudinal ventral kinety. *Antestrombidium* gen. nov. is distinguished by tripartite somatic kineties (circular and ventral kineties plus dextrally spiraled girdle kinety). *Sinistrostrombidium* and *Antestrombidium* branched separately from one another in phylogenetic trees, clustering with different clades of strombidiids. The new genera added to the diversities of ciliary patterns and small subunit rRNA gene sequences in strombidiids leads to presentation of a new hypothesis about evolution of the 12 known strombidiid genera, based on ciliary pattern and partly supported by molecular evidence. In addition, our new morphological and molecular analyses support establishment of a new order Lynnellida ord. nov., characterized by an open adoral zone of membranelles without differentiation of anterior and ventral membranelles, for *Lynnella*, but we remain unable to assign the genus to a subclass with confidence.

## Introduction

Oligotrich ciliates are common marine microplankters that usually contribute to the energy flow of the microbial loop [1–3]. More than 100 species of oligotrichs in 15 genera have been reported in marine and brackish water, and recent investigations demonstrated that the group is much more divergent than previously realized [4–11].

The family Strombidiidae is the major group of oligotrichs, characterized by a three-part oral ciliature (endoral membrane, anterior and ventral membranelles) and the stomatogenesis occurring within a transient tube [12, 13]. The somatic ciliature of strombidiids typically consists of only girdle (GK) and ventral (VK) kineties; however, an impressive diversity of ciliary patterns has originated from these two kineties and is regarded as important generic characters. Since Claparède & Lachmann [14] erected the well-known type-genus *Strombidium*, ten other strombidiid genera have been described [8, 12, 15–20]. The rich diversity of ciliary patterns in the family Strombidiidae raises questions about whether some patterns developed convergently and how they evolved. Evolutionary relationships of the main ciliary patterns of oligotrichs have been outlined in previous studies, based on purely morphological data [12, 19, 21]. However, hypotheses suggested by these studies were broadly descriptive of evolutionary lineages within the subclass Oligotrichia rather than elucidating specific patterns within the family Strombidiidae.

In the present paper, we describe two new species of oligotrichs that were isolated from coastal waters of southern China. All available evidence indicates that they represent two new genera in the family Strombidiidae, and their phylogenetic positions are analyzed using both morphological characters and SSrDNA trees. The new genera add to the diversity of ciliary patterns known in strombidiids and provide an opportunity for a better analysis of evolutionary relationships within the Strombidiidae. The phylogenetic position of the enigmatic genus *Lynnella* is also reconsidered based on new evidence.

## Materials and Methods

### Sample Collection, Observation and Terminology

Samples were collected from an interitdal zone and a mangrove wetland in 250 ml, wide-mouth bottles. The sampling locations are pulbic areas, thus no specific permissions were required to collect the materials necessary for the present study. No known endangered or protected species were involved in the present study.

**Fig 1:**
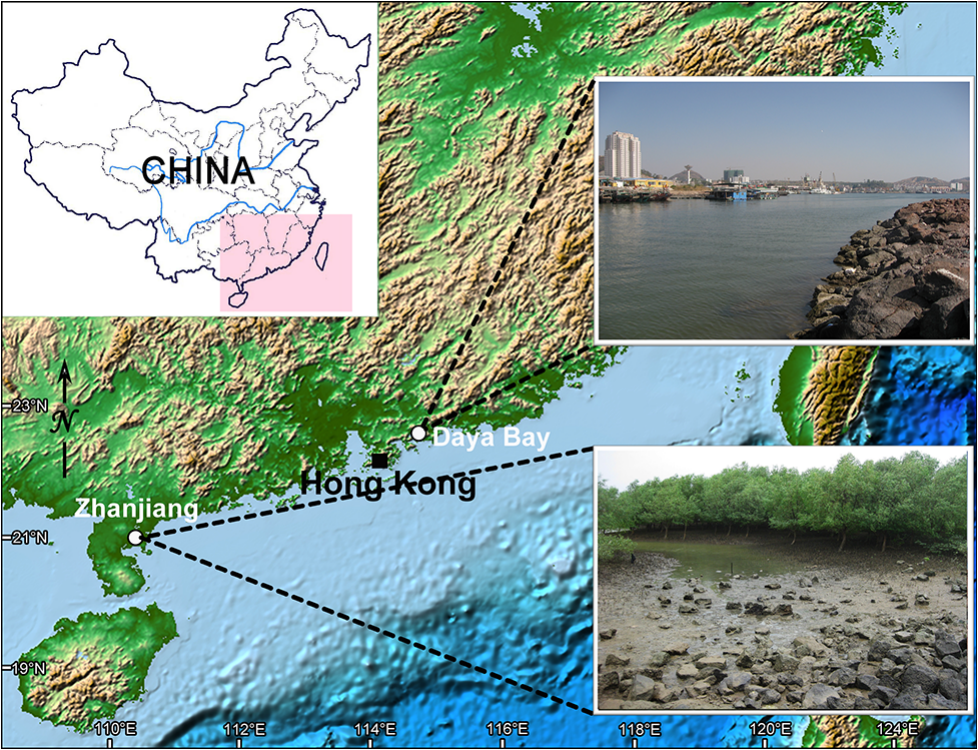
Location and habitats of sampling sites.

*Sinistrostrombidium cupiformum* gen. nov., sp. nov. was isolated from the littoral zone of Daya Bay (22°71′N; 114°54′E), Guangdong Province, China, on 27 May 2009 (Fig 1). The water temperature was 27.8°C, salinity 31.0 psu, and pH 8.6. *Antestrombidium agathae* gen. nov., sp. nov. was discovered in a mangrove wetland near Zhanjiang (21°36′N; 110°43′E), Guangdong Province, China, on 26 March 2010 (Fig 1). The water temperature was 19.7°C, salinity 23.9 psu, and pH 7.8.

The samples were transferred to Petri dishes (9 cm across; water depth 1 cm). The behaviour of the species was observed at about 20°C and then specimens were immediately isolated under a stereo microscope (Guiguang XTL-200, China) for further study in the laboratory. No cultures were established. Isolated living specimens were examined using bright field and differential interference contrast microscopy (Nikon Eclipse-80i, Tokyo, Japan). Staining with protargol according to the method of Wilbert [22] was used to reveal the infraciliature and nuclear apparatus. Counts and measurements on protargol-impregnated cells were performed at 1,000× magnification; in vivo measurements were made on ten cells for each species at 40–1,000× magnification. All measurements were done with a filar micrometer. Drawings of live specimens were based on direct observation and photomicrographs of ten cells for each species. Drawings of protargol-stained cells were made with a camera lucida at 1,000× magnification. Terminology follows Agatha [23], in which orientation of the somatic dikinetids is determined by whether the anterior or posterior kinetosome is ciliated, and classification follows Lynn [13].

### Extraction, Amplification and Sequencing of DNA

For each species, five cells were collected and rinsed according to the protocol of Xu *et al*. [24]. Genomic DNA was extracted with REDExtract-N-Amp Tissue PCR Kit (Sigma, St. Louis). The PCR was performed according to Liu *et al*. [25] with the universal eukaryotic primers EukA (5'-AACCTGGTTGATCCTGCCAGT-3') and EukB (5'-TGATCCTTCTGCAGGTTCACCTAC-3') [26]. The PCR product was purified with a TIAN gel Midi Purification Kit (Tiangen Bio. Co., Shanghai, China) and inserted into a pUCm-T vector (Sangon Bio. Shanghai, China). DNA from plasmids was harvested using a Mini-prep Spin Column Kit (Sangon) and sequenced by the Invitrogen sequencing facility (Shanghai, China).

### Phylogenetic Analysis

The SSrRNA gene sequences of all ciliates used in this study are available from the GenBank database. The SSrRNA gene sequences were aligned with Clustal X 1.83 [27] and the variable regions that could not be aligned unambiguously were removed manually from the initial alignment using Bioedit 7.0 [28], leaving a final alignment of 1,556 characters that was used to construct phylogenetic trees. The GTR + I + G evolutionary model was selected by both MrModeltest v.2 [29] and Modeltest v.3.7 [30] under the Akaike Information Criterion and used for Bayesian inference (BI) and maximum likelihood (ML) analyses, respectively. Bayesian trees were constructed with MrBayes 3.1.2 [31]. Four simultaneous chains were run for 1,500,000 generations, with sampling every 100 generations, and the first 3,750 (25%) trees discarded as burn-in. All remaining trees were used to calculate posterior probabilities using a majority rule consensus. The ML tree was constructed with PhyML v2.4.4 [32], including bootstrapping (1,000 replicates). A Maximum parsimony (MP) tree incorporating 1,000 bootstrap replicates was constructed with PAUP* 4.0b 10 [33] using a heuristic search with all characters coded as unordered. Phylogenetic trees were visualized with TreeView v1.6.6 [34] and MEGA 4.0 [35].

PAUP* 4.0b 10 was used to generate constrained ML trees under the GTR + I + G model to test the hypothesis that *Lynnella* clusters with subclass Oligotrichia and Choreotrichia, respectively. The best constraint trees (i.e. with lowest-lnL values) were compared with unconstrained ML trees using the approximately unbiased (AU) and Shimodaira-Hasegawa (SH) tests [36] implemented in CONSEL v0.1i [37]. Similarities and pairwise distances between SSrRNA gene sequences of *Sinistrostrombidium cupiformum* gen. nov., sp. nov., *Antestrombidium agathae* gen. nov., sp. nov. and typical species of other strombidiid genera were analysed using Bioedit 7.0.

### Nomenclatural Acts

The electronic edition of this article conforms to the requirements of the amended International Code of Zoological Nomenclature, and hence the new names contained herein are available under that Code from the electronic edition of this article. This published work and the nomenclatural acts it contains have been registered in ZooBank, the online registration system for the ICZN. The ZooBank LSIDs (Life Science Identifiers) can be resolved and the associated information viewed through any standard web browser by appending the LSID to the prefix "http://zoobank.org/". The LSID for this publication is: urn:lsid:zoobank.org:pub: 0240D079-D523-4AE5-8639-D127609D18EA. The electronic edition of this work was published in a journal with an ISSN, and has been archived and is available from the following digital repositories: PubMed Central, CLOCKSS.

## Results

### Morphological Descriptions

Class Spirotrichea Bütschli, 1889

Subclass Oligotrichia Bütschli, 1887

Order Strombidiida Petz & Foissner, 1992

Family Strombidiidae Fauré-Fremiet, 1970

Genus *Sinistrostrombidium* gen. nov. urn:lsid:zoobank.org:act: 3C0AC3A7-5A5F-438A-96A9-00139B065581

Diagnosis: Members of Strombidiidae with ventral kinety and sinistrally spiraled girdle kinety; oral primordium develops below left end of girdle kinety.

Etymology: Composite of the Latin *sinistro-* (left) and the generic name *Strombidium*, referring to the sinistrally spiraled girdle kinety and the general similarity to the genus *Strombidium* with respect to other characters. Neuter gender.

Type species: *Sinistrostrombidium cupiformum* gen. nov., sp. nov. urn:lsid:zoobank.org:act: 8F5C8BD0-B8A3-4405-B6E8-8AE6DFE5AC3A

**Fig 2:**
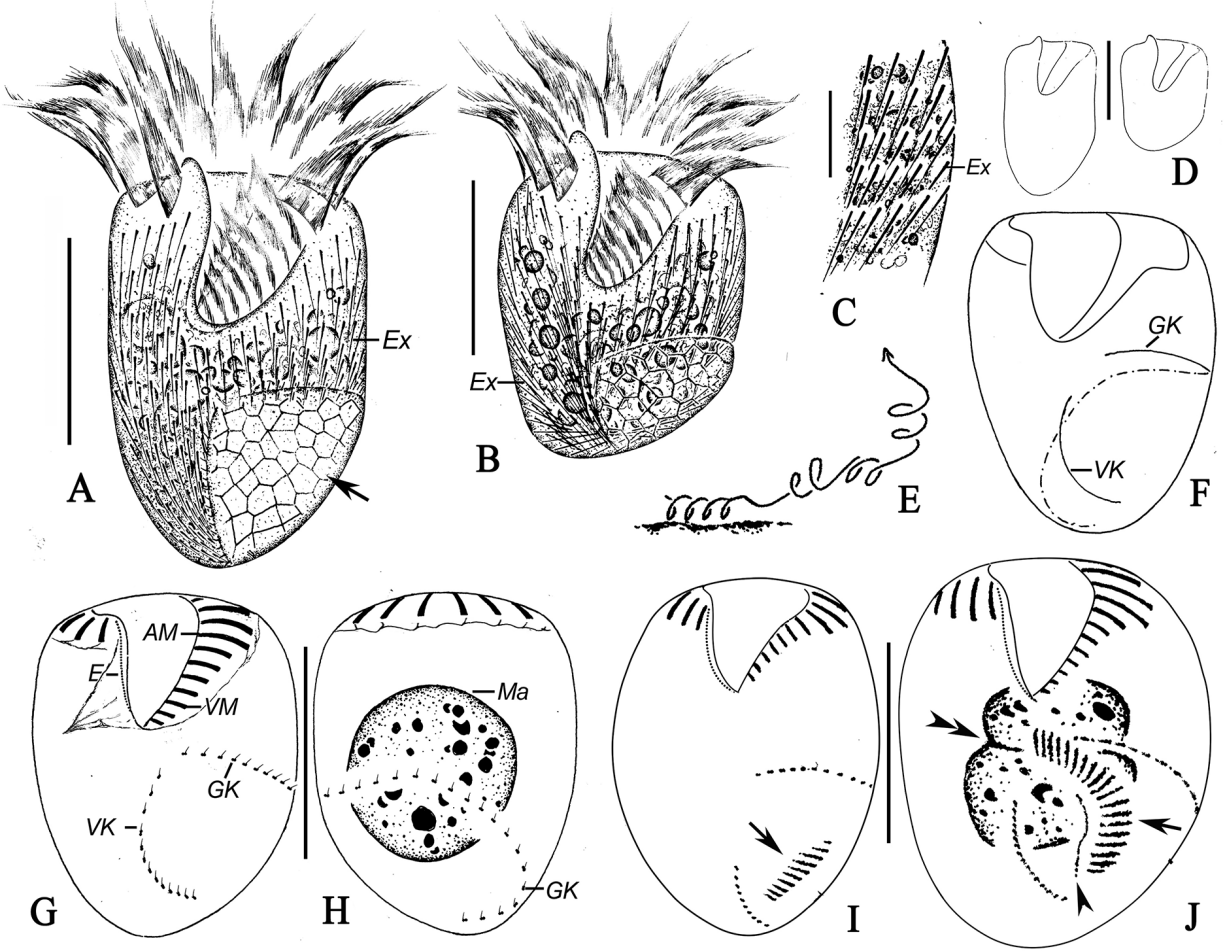
*Sinistrostrombidium cupiformum* gen. nov., sp. nov. from life (A-E) and after staining with protargol (F-J). A, B. Ventral view of different specimens; arrow marks the cortical platelets; C. Detailed view showing the distribution of extrusomes; D. Variations in shape of the body; E. Swimming trace; F. Pattern of somatic ciliature; G, H. Ventral (G) and dorsal (H) views of the same specimens showing the ciliary pattern and macronuclear nodules; I, J. Ventral views of cells in the early (I) and middle (J) stages of division, arrows indicate the oral primordium, arrowhead marks the new endoral membrane, and double-arrowheads note the replication band of the macronucleus. Legend: AM-anterior membranelles; E-endoral membrane; Ex-extrusomes; GK-girdle kinety; Ma-macronucleus; VK-ventral kinety; VM-ventral membranelles. Scale bars: A, B, D, G, H. 20 μm; C. 5μm.

**Fig 3:**
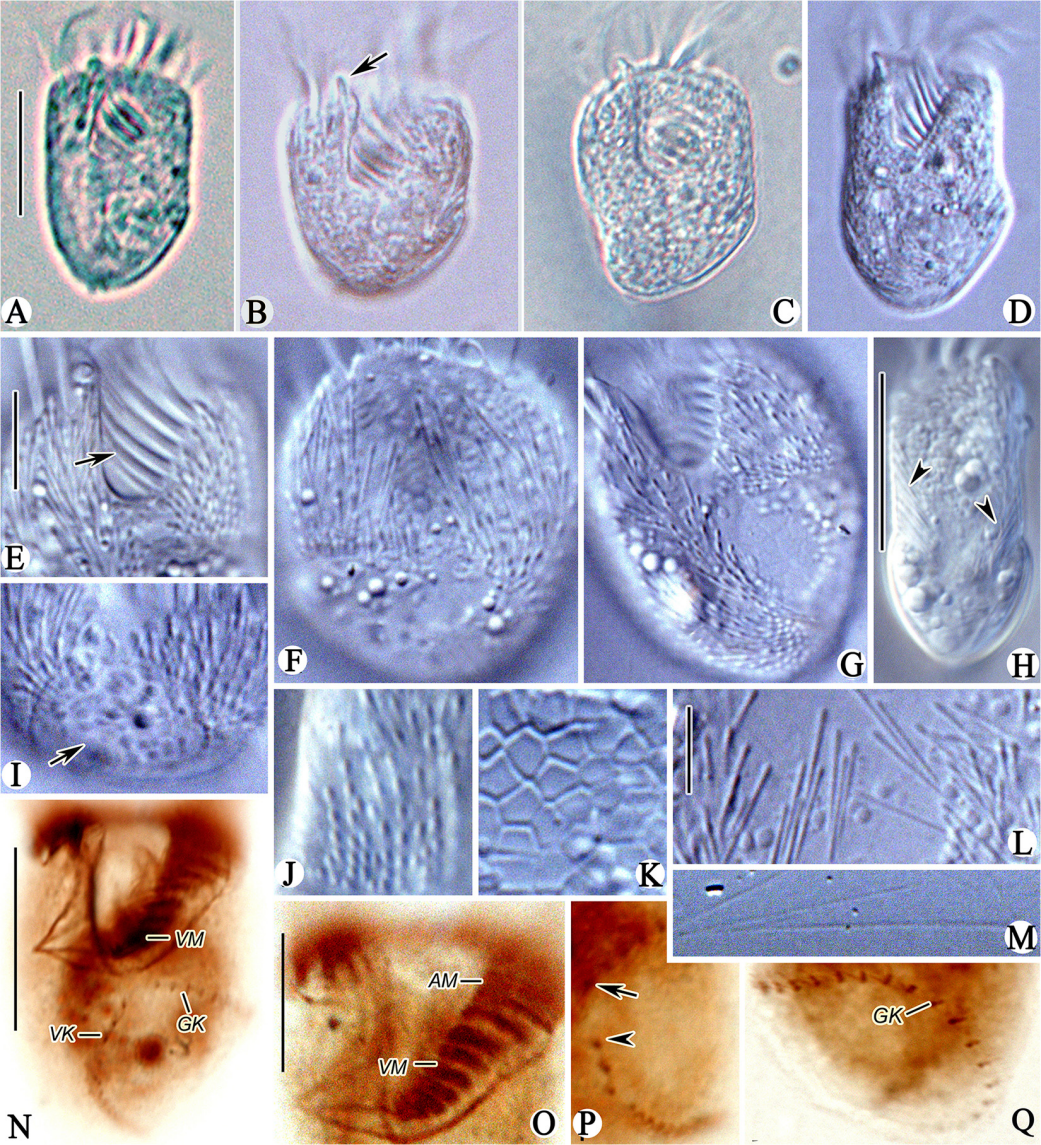
Photomicrographs of *Sinistrostrombidium cupiformum* gen. nov., sp. nov. from life (A-M) and after staining with protargol (N-Q). A. Ventral view of a typical individual; B-D. Variation in shape of the body, note the apical protrusion (arrow); E. Ventral view of anterior part of cell showing the VM (arrow); F-H. Dorsal (F), ventral (G) and lateral (H) views showing the distribution of extrusomes (arrowheads); I. Ventral view of posterior part of cell showing the hemitheca (arrow); J. Outer ends of extrusomes; K. Detail of hemitheca with polygonal cortical platelets; L. Undischarged extrusomes; M. Discharged extrusomes; N. Ventral view showing the somatic kineties and AZM; O. Ventral view of anterior part of cell showing the AM and VM; P. Ventral view, arrowhead marks the anterior end of VK, arrow indicates the anterior end of GK; Q. Dorsal view showing the GK. Legend: AM-anterior membranelles; GK-girdle kinety; VK-ventral kinety; VM-ventral membranelles. Scale bars: A-D, H, N. 20 μm; E-G, I, O-Q. 10 μm; J-M. 5μm.

*Sinistrostrombidium cupiformum* gen. nov., sp. nov. (Figs 2 and 3; Table 1)

**Table 1:** Morphometric data of *Sinistrostrombidium cupiformum* gen. nov., sp. nov. (first row) and *Antestrombidium agathae* gen. nov., sp. nov. (second row).

Characters	Min	Max	Mean	SD	n
Length of cell (μm)	27	34	30.3	2.20	20
	41	51	45.1	3.36	11
Width of cell (μm)	20	28	24.4	1.98	20
	21	29	24.1	2.74	11
Distance from anterior end to cytostome (μm)	12	14	13.3	0.80	20
	15	21	17.3	2.06	10
Length of ventral kinety (μm)	8	12	10.5	1.51	13
	6	11	9.14	1.77	7
Number of anterior membranelles	11	13	11.9	0.49	20
	15	21	18.1	1.87	11
Number of ventral membranelles	7	8	7.6	0.51	20
	7	9	7.8	0.72	12
Number of dikinetids in girdle kinety	24	31	27.2	1.96	13
	37	52	43.3	3.86	10
Number of dikinetids in circular kinety	—	—	—	—	—
	25	34	28.5	2.58	11
Number of dikinetids in ventral kinety	9	14	11.8	1.54	13
	10	16	11.9	1.76	9
Diameter of macronucleus (μm)	14	17	15.6	0.94	17
	—	—	—	—	—
Length of macronucleus (μm)	—	—	—	—	—
	13	17	15.1	1.45	11
Width of macronucleus (μm)	—	—	—	—	—
	8	12	9.7	1.27	11

All data based on specimens stained with protargol; Max-maximum value observed; Mean-arithmetic mean; Min-minimum value observed; n-sample size; SD-standard deviation.—: data unavailable.

Diagnosis: Size approximately 40 × 30 μm for living cells and 30 × 25 μm after staining with protargol; body cupiform to broadly cylindroid. Hemitheca covering left side of posterior part of cell, and extrusomes scattered all over cell surface except area beneath hemitheca. Girdle kinety consisting of approximately 27 dikinetids, extending from center of ventral side to dorsal side, curving toward left and terminating at posterior end of cell. Ventral kinety consisting of approximately 12 dikinetids, curving slightly toward left side of cell and terminating at posterior end. Adoral zone of membranelles composed of approximately 12 anterior and 8 ventral membranelles. Single spheroidal macronucleus.

Etymology: The Latin word “*cupiformum*” refers to the shape of the body, which is generally cylindroid or barrel-shaped, with a rounded posterior end.

Type locality: Littoral zone of Daya Bay (22°71′N; 114°54′E), Guangdong, China.

Deposition of Type-specimens: One slide containing the protargol-stained holotype specimen was deposited at the Natural History Museum, London, with registry number NHMUK 2011.11.23.1. Two paratype slides (protargol preparations) were deposited in the collection of the Laboratory of Protozoology, Ocean University of China, with registry numbers LWW2009052704-01 and LWW2009052704-02

Gene sequence: A sequence of the SSrRNA gene of *S*. *cupiformum* has been deposited in the GenBank database with accession number JX310366.

Description: Living cells measured 35–45 × 20–35 μm and protargol-stained cells 27–34 × 20–28 μm. Shape of body slightly variable, generally cupiform to broadly cylindrical (Figs 2A, 2B and 2D; 3A–3D). Anterior end transversely truncated, with 3 μm-long apical protrusion at the right end of the peristome in living cells that is undetectable after fixation (Figs 2A and 2B; 3B–3D arrow). Posterior end bluntly rounded and slanted slightly to the right (Figs 2A, 2B; 3A and 3C). Subequatorial area usually widest. Cell dorsoventrally flattened, with thickness/width ratio of approximately 4/5 (Fig 3H).

Pellicle thin, left side of posterior half of cell covered by hemitheca composed of polygonal platelets approximately 2 μm in diameter (Fig 2A and 2B, arrows; 3F-I, arrow, K). Hemitheca covers left 2/3 of posterior half of dorsal side and left 1/2 of posterior half of ventral side. Hemitheca located posterior to GK, where cell surface is distinctly distended. Cytoplasm is colourless and usually filled with numerous food vacuoles measuring 2–3 μm in diameter (Fig 2B). Extrusomes distributed over all of cell surface except area beneath hemitheca in irregular, longitudinal rows of 5–15 individuals (Figs 2A and 2B; 3F, 3G and 3H arrowheads). Some extrusomes densely packed together near edge of hemitheca, but rest sparsely distributed in anterior portion of cell (Figs 2C; 3G, 3I and 3J). Extrusomes near edge of hemitheca transversely oriented and those in anterior part of cell oriented at a slightly oblique angle (Fig 3H, arrowheads). Undischarged extrusomes needle-like, measuring approximately 5 × 0.4 μm, with rounded anterior and sharply pointed posterior end (Fig 3L). Discharged extrusomes thread-like and approximately 35 μm long (Fig 3M). Macronucleus spheroidal and centrally located, measuring approximately 15 μm in diameter after staining with protargol (Fig 2H). Micronucleus, contractile vacuole and cytopyge not observed. In Petri dish with *in situ* water at room temperature, cells always swimming in spirals (about 50 μm across) by rotating around their longitudinal axis with sudden changes in direction when disturbed (Fig 2E).

Somatic cilia arranged as girdle (GK) and ventral kineties (VK) (Figs 2G and 2H; 3Q). Girdle kinety composed of 24–31 dikinetids and following sinistrally directed, helical path around posterior half of cell; GK extending from center of ventral side below the oral cavity, across left ventral and dorsal sides, slanting toward posterior end, and curving toward left to terminate at posterior end of cell (Figs 2F–2H; 3Q). Left kinetosome of each dikinetid in GK bears short, cylindroid cilium measuring approximately 2 μm long. Ventral kinety extends from posterior 1/3–1/4 of cell, curves slightly toward left side, and terminates at posterior end of cell (Figs 2F and 2G; 3N and 3P). Ventral kinety composed of 9–14 densely arranged dikinetids, 2–3 of which are usually widely spaced at anterior end (Fig 2G). Each dikinetid of VK possesses one short cilium, approximately 2 μm long, on anterior kinetosome.

Oral apparatus occupies entire anterior end of cell. Buccal field wide and deep, extending obliquely for 2/5 of length of cell (Figs 2A and 2B; 3D and 3E). Adoral zone of membranelles (AZM) composed of 11–13 anterior (AM) and 7–8 ventral membranelles (VM) (Figs 2G; 3O). Anterior membranelles extend from right side of peristome below apical protrusion, around dorsal rim of peristome, to join with VM on left side of peristome (Figs 2G; 3N and 3O). Cilia of AM approximately 18–22 μm long in living cells, with tips splayed obliquely outward in swimming cells (Fig 2A and 2B). Cilia of VM approximately 3–5 μm in length. Bases of AM approximately 5 μm long and distinctly longer than those of VM, which gradually shorten from upper to lower (approximately 2–3 μm) (Figs 2G; 3O). All membranelles comprise three rows of basal bodies except for two posteriormost membranelles, which probably comprise only two rows. No thigmotactic membranelle observed. Endoral membrane approximately 7 μm long, located on inner wall of buccal cavity, and apparently composed of single row of kinetosomes (Fig 2G). System of argentophilic fibres developed and associated with adoral membranellar zone. Pharyngeal fibres up to 5 μm long extend obliquely rightward (Figs 2G and 2H; 3N).

Two divisional stages observed. One cell in early stages of division formed oral primordium left of VK and below anterior end of GK (Fig 2I, arrow). Another cell in mid- to-late stages of division developed oral primordium further toward posterior half of cell at point in time when posterior portion of newly built AZM bends to right and anterior part passes through gap between the GK and VK (Fig 2J, arrow). In this cell, endoral membrane could be seen originating to right of new AZM (Fig 2J, arrowhead), and replication band was recognizable in center of macronuclear nodule (Fig 2J, double-arrowheads)

Genus *Antestrombidium* gen. nov. urn:lsid:zoobank.org:act: 5356F427-ACA8-4632-A26E-203C786B4599

Diagnosis: Members of Strombidiidae with three somatic kineties—i.e., dextrally spiraled girdle kinety, circle kinety and ventral kinety.

Etymology: Composite of the Latin *Ante*- (before) and the generic name *Strombidium*. Neuter gender.

Type species: *Antestrombidium agathae* gen. nov., sp. nov. urn:lsid:zoobank.org:act: 1B630D57-DD65-488B-8C39-FD0A44F2C6FE

**Fig 4:**
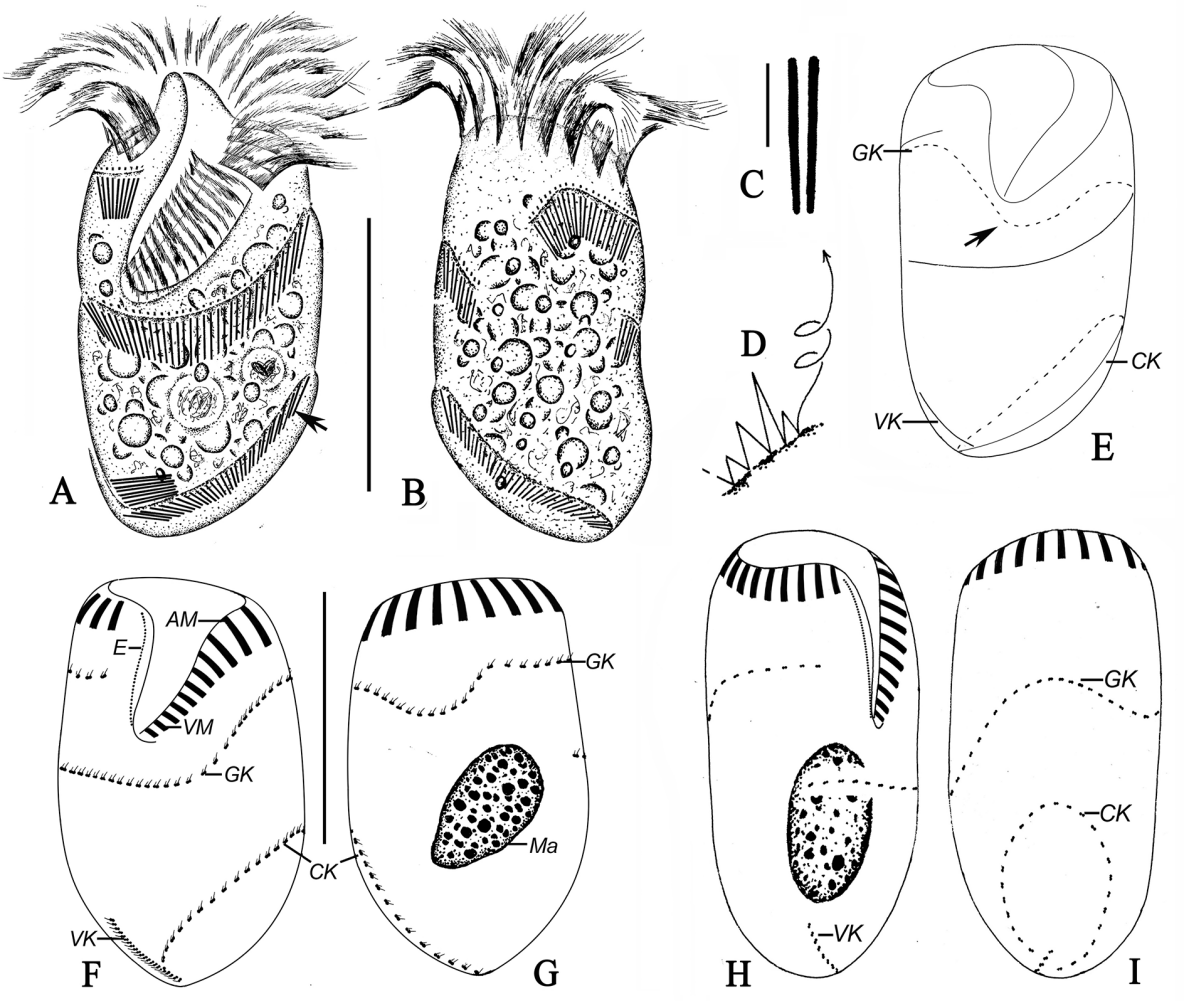
*Antestrombidium agathae* gen. nov., sp. nov. from living observations (A-D) and after staining with protargol (E-I). A, B. Ventral and dorsal views of a typical specimen, arrows mark the extrusomes; C. Undischarged extrusomes; D. Swimming trace; E. Pattern of somatic ciliature; F, G. Ventral (F) and dorsal (G) views of the same specimen showing the ciliary pattern and macronucleus; H, I. Right (H) and left (G) lateral views showing the ciliary pattern. Legend: AM-anterior membranelles; CK-circular kinety; E-endoral membrane; GK-girdle kinety; Ma-macronucleus; VK-ventral kinety; VM-ventral membranelles. Scale bars: A, B, E-I. 30 μm; C. 3 μm.

**Fig 5:**
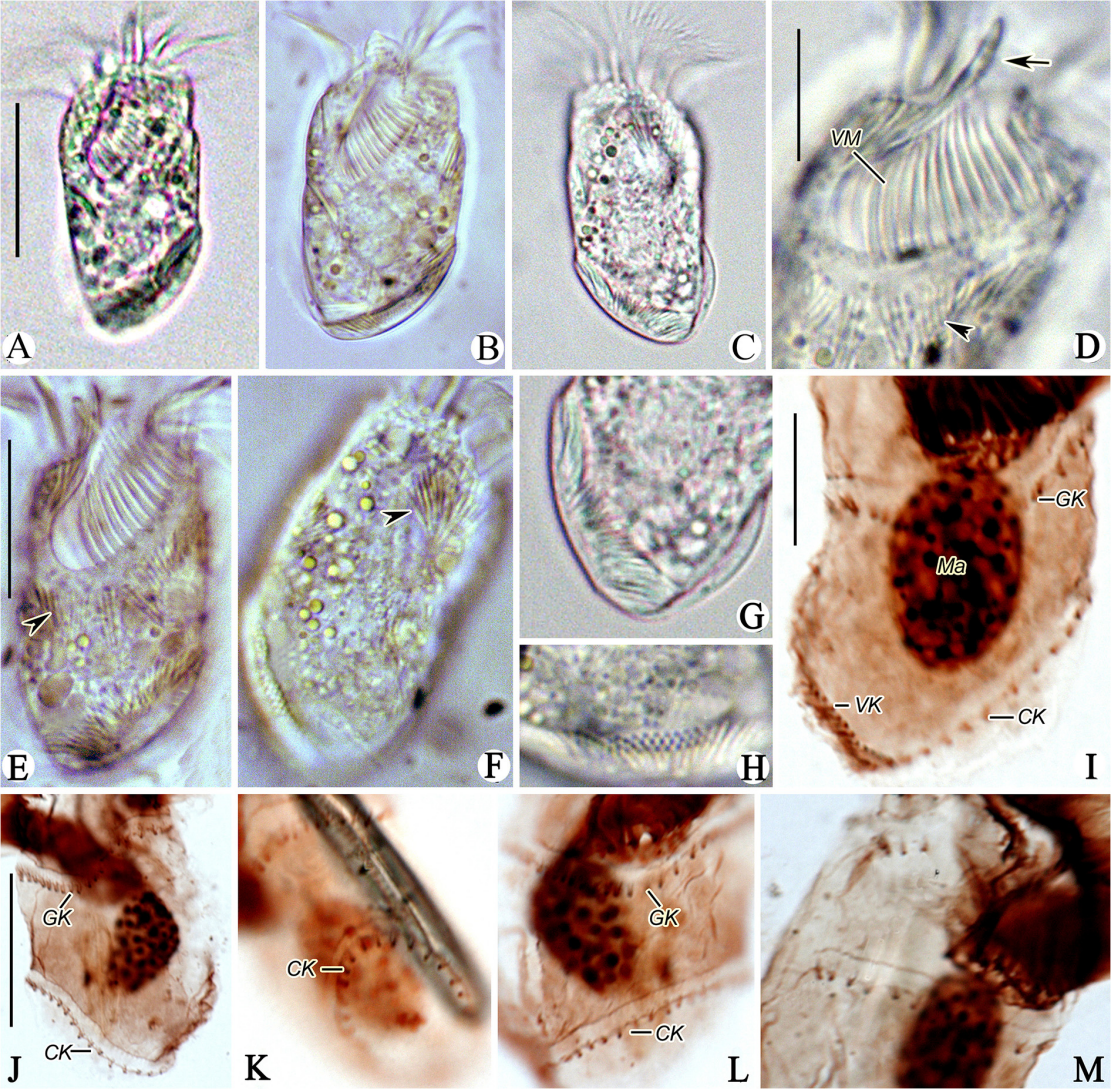
Photomicrographs of *Antestrombidium agathae* gen. nov., sp. nov. from life (A-H) and after staining with protargol (I-M). A, B. Ventral views of two individuals; C. Dorsal view; D. Ventral view of anterior part of cell, arrow marks the apical protrusion and arrowhead indicates the extrusomes; E, F. Ventral (E) and Dorsal (F) views showing the distribution of extrusomes (arrowheads); G, H. Posterior portion of cell showing the distribution of extrusomes; I, J. Ventral and dorsal views of posterior portion of cell, showing the somatic kineties; K, L. Left and ventral views showing the GK and CK; M. Right lateral view showing the GK. Legend: CK-circular kinety; GK-girdle kinety; Ma-macronucleus; VK-ventral kinety; VM-ventral membranelles. Scale bars: A-C. 30 μm; D, G-I, L, M. 10 μm; E, F, J, K. 20 μm.

*Antestrombidium agathae* gen. nov., sp. nov. (Figs 4 and 5; Table 1)

Diagnosis: Cell size measure approximately 55 × 25 μm in vivo and 45 × 25 μm after protargol-stained; body elliptical, with obvious apical protrusion. Extrusomes arranged in closely spaced series alongside somatic kineties. Girdle kinety making one helical circuit around cell following dextral path, composed of approximately 43 dikinetids; circle kinety surrounding left posterior part of cell, composed of approximately 29 dikinetids; ventral kinety extending obliquely across right posterior part of ventral surface of cell, composed of approximately 12 dikinetids. Adoral zone of membranelles consisting of approximately 18 anterior and 8 ventral membranelles. Single ovoid macronucleus.

Dedication: We dedicate this new species to Dr. Sabine Agatha, University of Salzburg, Austria, in recognition of her significant contributions to knowledge of oligotrich and choreotrich ciliates.

Type locality: Brackish water from a mangrove wetland near Zhanjiang (21°36′N; 110°43′E), Guangdong, China.

Deposition of Type-specimens: One slide containing the protargol-stained holotype specimen and paratype specimens was deposited in the Laboratory of Protozoology, Ocean University of China, with registry number LWW2010032606.

Gene sequence: A sequence of the SSrRNA gene of *A*. *agathae* has been deposited in the GenBank database with accession number JX310365.

Description: Living **c**ells measured 50–60 × 20–30 μm and protargol-stained specimen 41–51 × 21–29 μm. Body elliptical, with anterior end truncated and right end of peristome projecting to form anterior protrusion approximately 4 μm high and undetectable after fixation. Posterior end of cell tapered slightly and tilted toward right (Figs 4A and 4B; 5A–5C).

Cells usually dark at low magnifications owing to presence of numerous food granules in the cytoplasm that are 2–3 μm across (Fig 4A and 4B). Cytoplasm colourless and apparently lacking cortical platelets. Extrusomes closely spaced to form bands along GK, VK and circle kinety (CK). Their attachment sites arranged in one row above GK, VK and CK, where cell surface is elevated to form conspicuous ridges (Fig 4A and 4B, arrow; 5D-H, arrowheads). Undischarged extrusomes rod-shaped, measuring approximately 6 × 0.6 μm (Fig 4C). Macronucleus ovoid, located in posterior half of cell, and measuring approximately 10 × 15 μm after staining with protargol (Figs 4G and 4H; 5I). Micronucleus, contractile vacuole and cytopyge not observed. In Petri dish with *in situ* water at room temperature, cell normally moves by crawling over debris but swims away by rotation about longitudinal axis in spirals (about 70 μm across) when disturbed (Fig 4D).

Somatic ciliature composed of the following three parts: GK, CK, and VK (Fig 4E). Girdle kinety, consisting of 37–52 dikinetids, curves toward posterior on dorsal side and makes one helical circuit around cell in dextral direction. GK extends from right end of AZM, crosses dorsal surface of cell, continues around left side, and crosses ventral surface to point almost directly posterior to its anterior end (Figs 4E–4I; 5I–5M). Circular kinety consisting of 25–34 dikinetids, occupying posterior 1/3 of left side of cell and forming circle with small gap in posterior end of cell through which one end of VK passes (Figs 4E and 4I; 5J–5L). Both kinetosomes of each dikinetid in GK and CK bear fusiform cilium, with left (anterior) one usually longer (approximately 3 μm long) than right (posterior) one (approximately 1 μm long) (Fig 5M). Ventral kinety located on posterior portion of right side of cell, extending from gap in CK along right side to posterior 1/5 of cell (Figs 4E, 4F and 4H; 5I). Ventral kinety composed of 10–16 dikinetids, with left (anterior) kinetosome of each dikinetid bearing short cilium approximately 2 μm long.

Buccal field conspicuously broad and deep, obliquely spanning 2/5 of length of cell (Figs 4A; 5A and 5C). Adoral zone of membranelles consists of 15–21 AM and 7–9 VM, which are continuous (Fig 4F). Cilia of AM approximately 15–20 μm long in living cells, splayed outward horizontally when cell is swimming (Figs 4A; 5B). Cilia of VM approximately 3–5 μm long. Bases of AM slightly longer (approximately 6 μm) than those of VM (approximately 4 μm) (Fig 4F). All membranelles comprise three kinety rows, except for two posteriormost membranelles which probably comprise only two rows. Endoral membrane on inner wall of buccal cavity extends to center of apical protrusion, approximately 10 μm long, apparently composed of single row of kinetosomes (Fig 4F). Pharyngeal fibres and thigmotactic membranelle not observed.

### Molecular Phylogenetics

**Fig 6:**
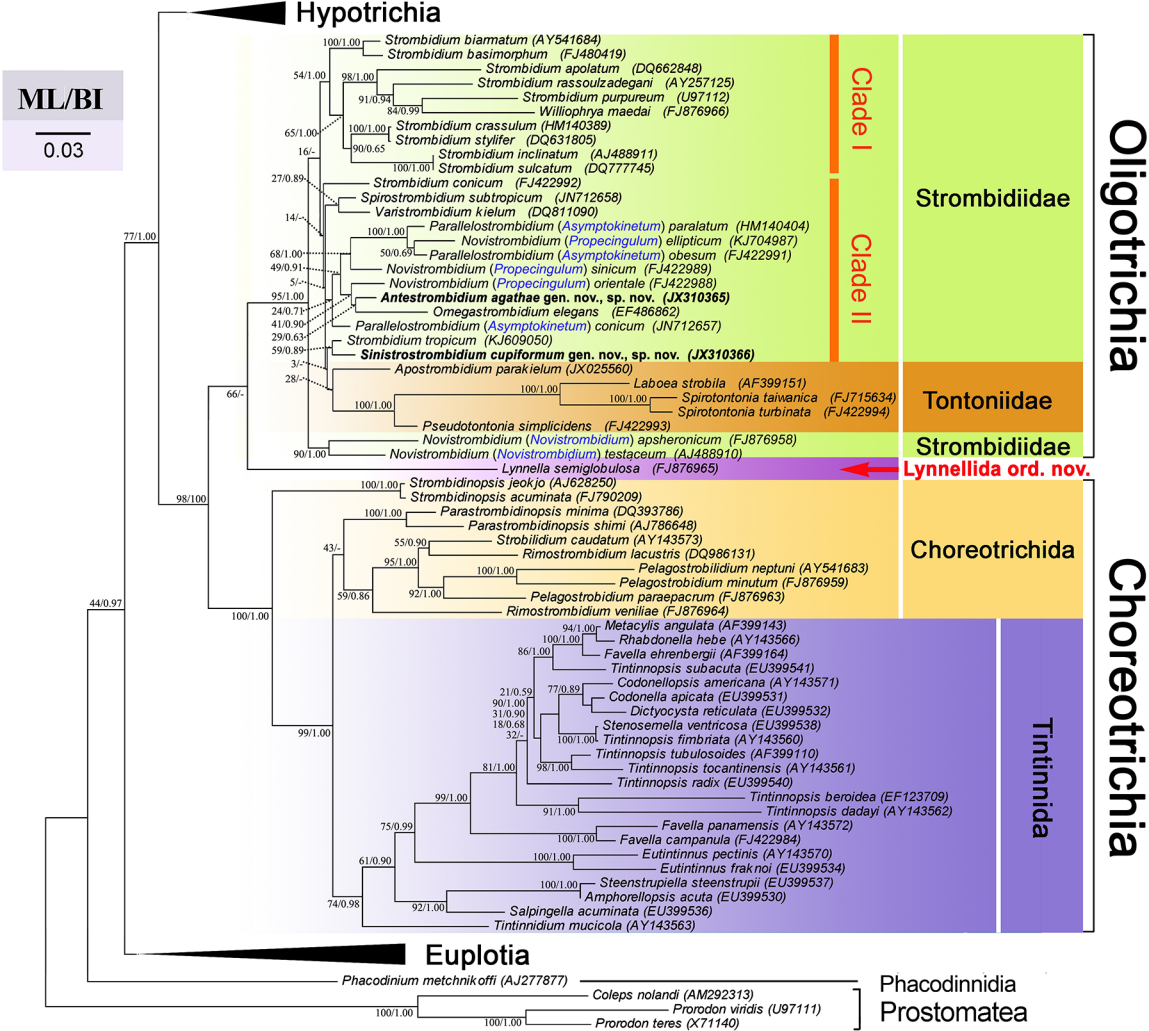
Maximum Likelihood tree inferred from SSrRNA gene sequences. Numbers at the nodes are support values presented in the following order: Maximum Likelihood (ML) bootstrap values and Bayesian inference (BI) posterior probabilities. Nodes absent from BI tree are indicated by a hyphen. The scale bar indicates the number of substitutions per 10 nucleotides.

The SSrRNA gene sequences of *Sinistrostrombidium cupiformum* and *Antestrombidium agathae* were deposited into GenBank with accession numbers JX310366 and JX310365, respectively. In all our phylogenetic trees (Fig 6), monophyly of the subclasses Oligotrichia and Choreotrichia was supported strongly at their basal nodes, respectively (95% ML, 1.00 BI; 100% ML, 1.00 BI). *Lynella semiglobosa* branches basally off the Oligotrichia with low support (66% ML).

In the oligotrich clade, *Novistrombidium apsheronicum* and *N*. *testaceum* are basal to all other members of the clade. Most species of *Strombidium* form a poorly resolved assemblage (clade I, Fig 6) comprising three relatively well-supported subclades, with a clade consisting of *S*. *basimorphum* and *S*. *biarmatum* occupying a basal position. *Williophrya maedai* nests within one of these subclades (Fig 6). All other strombidiid species, together with tontoniids, form a second, poorly supported assemblage (clade II, Fig 6), consisting of four subclades. *Strombidium conicum* does not cluster with its congeneric species but branches at the base of the clade II, representing the first subclade. *Spirostrombidium subtropicum* and *Varistrombidium kielum* branch separately, forming the second subclade. *Sinistrostrombidium cupiformum* gen. nov., sp. nov. clusters with *Strombidium tropicum* on an isolated basal branch that is an unresolved sister to the poorly supported subclade containing tontoniids and *Apostrombidium parakielum*. The fourth subclade is composed of three groups. *Antestrombidium agathae* gen. nov., sp. nov. associates with *Omegastrombidium elegans*, which then clusters with *Novistrombidium orientale* (Fig 6). This group is sister to a poorly supported group containing *Parallelostrombidium paralatum*, *P*. *ellipticum*, *P*. *obesum* and *N*. *sinicum*. *Parallelostrombidium conicum* represents the third group in this subclade (Fig 6).

## Discussion

### Comparison of *Sinistrostrombidium* and *Antestrombidium* to Other Strombidiid Genera

The arrangement and composition of somatic kineties are regarded as important diagnostic characters of strombidiid genera [12, 23]. Ten genera are presently in the family Strombidiidae (Table 2) [8, 11, 12, 17–20].

**Table 2:** Morphological comparison of somatic kineties among the known genera in the family Strombidiidae.

Genus	Number of somatic kinety fragments	Pattern of girdle or other kineties	ventral kinety	Source of data
*Strombidium*	1 or 2	Transverse	present	[18]
*Novistrombidium*	2	Dextrally spiraled	present	[17]
*Spirostrombidium*	2 or 3	Dextrally spiraled, with posterior portion of GK recurved and inversely parallel to VK	present	[12]
*Parallelostrombidium*	2	Dextrally spiraled, with posterior portion of GK parallel to VK	present	[12]
*Omegastrombidium*	2 or 3	Ω-shaped	present	[12]
*Apostrombidium*	2 or 3	Curved to posterior end of cell on both ventral and dorsal sides	absent	[11]
*Varistrombidium*	5	Obliquely and parallel across ventral side, with longest two extending to posterior end of dorsal side	absent	[20]
*Opisthostrombidium*	2	Oral primordium anterior to GK and extrusome stripe	present	[19]
*Foissneridium*	2	Oral primordium between GK and extrusome stripe	present	[19]
*Williophrya*	2	Horizontally oriented and bipartite	absent	[8]
*Sinistrostrombidium gen*. *nov*.	2	Sinistrally spiraled	present	Present work
*Antestrombidium gen*. *nov*.	3	Dextrally spiraled GK and circular CK	present	Present work

CK-circle kinety; GK-girdle kinety; VK-ventral kinety;

*Sinistrostrombidium* gen. nov. can be distinguished easily from *Novistrombidium*, *Parallelostrombidium*, and *Spirostrombidium* by its sinistrally directed, helical GK, compared to the dextrally helical GKs of the latter genera [12, 17]. The GKs of *Foissneridium*, *Omegastrombidium*, *Opisthostrombidium*, and *Strombidium* are transversely circular or Ω-shaped rather than helical, and thus separate them from *Sinistrostrombidium* [12, 19]. *Williophrya*, *Apostrombidium*, and *Varistrombidium* have a GK that is split into two or more parts; thus, all three differ in a fundamental way from *Sinistrostrombidium* with a complete GK [8, 18, 20].

The pattern of the GK in *Sinistrostrombidium* represents a unique morphological novelty among members of the family Strombidiidae and, therefore, justifies establishment of the new genus. Furthermore, the validity of *Sinistrostrombidium* is supported by the fact that it branches separately from other strombidiid genera in our phylogenetic analyses, and its SSrRNA sequence shows relatively low similarity (93.0–97.8%) to and high pairwise distance (0.020–0.066) from those of other strombidiid species (Table 3).

**Table 3:** Similarities (first row) and pairwise distances (second row) between SSrRNA gene sequences of *Sinistrostrombidium cupiformum* gen. nov., sp. nov., *Antestrombidium agathae* gen. nov., sp. nov. and typical species of other strombidiid genera.

Species	1	2	3	4	5	6	7
*S*. *cupiformum*	97.0%	93.0%	95.8%	97.6%	97.8%	95.6%	97.5%
	0.026	0.066	0.038	0.021	0.020	0.039	0.021
*A*. *agathae*	97.0%	93.4%	95.3%	97.6%	97.7%	96.4%	96.8%
	0.026	0.062	0.044	0.022	0.022	0.031	0.029

1, *Strombidium conicum*; 2, *Williophrya maedai*; 3, *Novistrombidium testaceum*; 4, *Spirostrombidium subtropicum*; 5, *Parallelostrombidium conicum*; 6, *Omegastrombidium elegans*; 7, *Varistrombidium kielum*.

The somatic kineties are composed of a GK and a VK or a GK alone in all strombidiid genera except *Varistrombidium* and *Antestrombidium* gen. nov. In the latter genus, the somatic kineties are distinctive by being differentiated into three parts: GK, VK and CK, a pattern that also differs markedly from the five somatic kineties of *Varistrombidium* [20]. Thus, *A*. *agathae* represents another morphological novelty within the family Strombidiidae, justifying establishment of a new genus for it. Likewise, its SSrRNA sequence shows relatively low similarity (93.4–97.6%) to and high pairwise distance (0.022–0.062) from those of other strombidiids (Table 3), which supports the validity of the new genus.

### Evolutionary Hypothesis of Strombidiids Based on Molecular and Morphological Evidence

**Fig 7:**
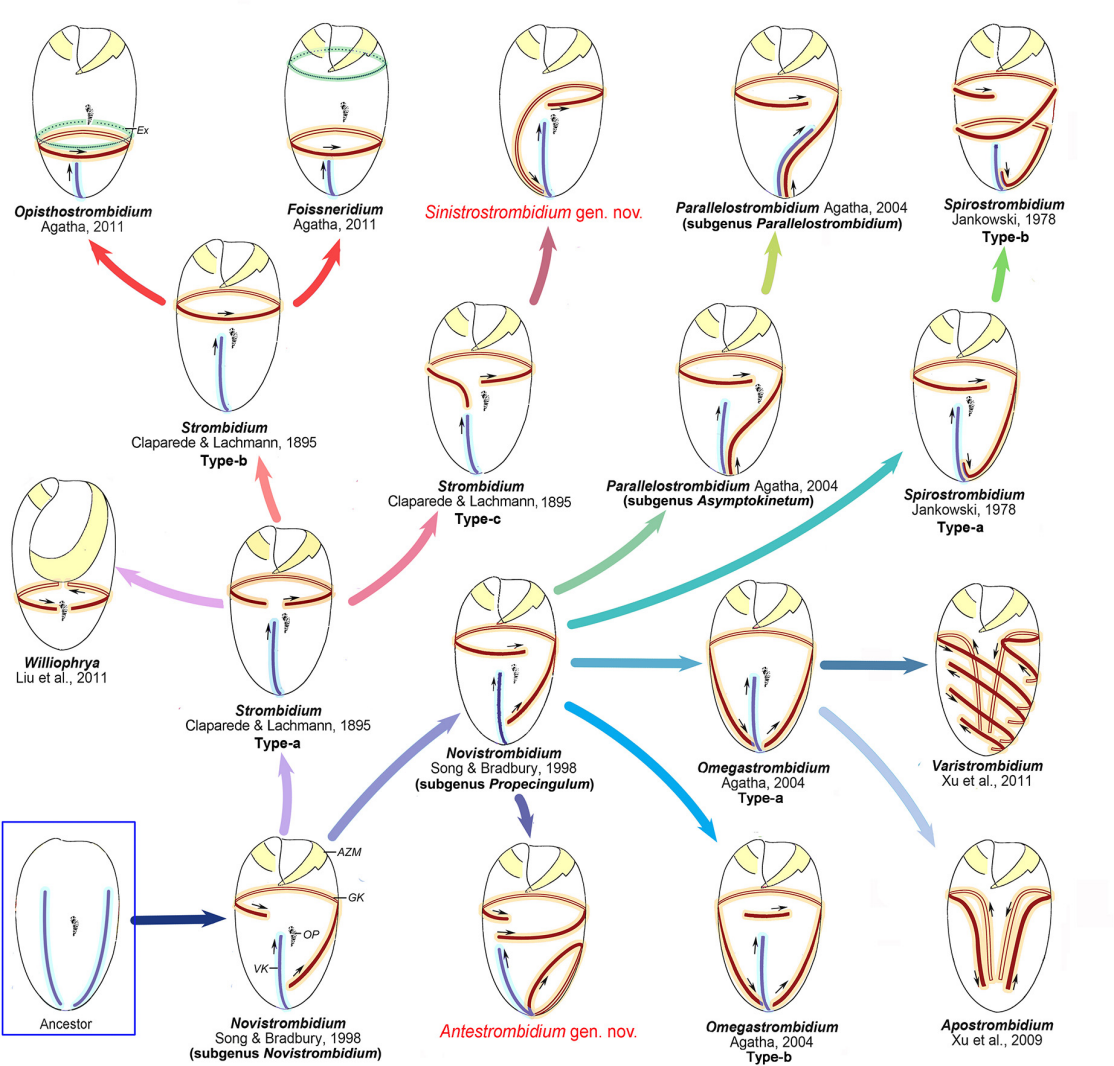
Hypothetical evolution of Strombidiidae based on an analysis of somatic ciliature. Small arrows mark the orientation of the kineties. Legend: AZM-adoral zone of membranelles; Ex-extrusomes; GK-girdle kinety; OP-oral primordium; VK-ventral kinety.

Up to now, 16 different ciliary patterns can be identified in 12 genera belonging to the family Strombidiidae (Fig 7, previous analyses suggested that *Laboea* should represent a member of family Tontoniidae rather than Strombidiidae and thus its ciliary pattern was not included here [21, 38, 39]) and SSrRNA gene sequences of 28 species of oligotrichs are available for analysis, which prompts us to offer an update of the evolutionary hypothesis of oligotrichs and relate it to morphotypes of strombidiids.

The ontogenetic process can provide important evidence to understand the evolution of ciliary patterns in oligotrichs [12]. However, the ontogenetic data of most strombidiids are not available at present. On the other hand, the differences of somatic kineties in the location, length, number and spiral extent, which reflect the different triggers of ciliary pattern development during the ontogenesis such as split, migration and extension, could be important clues to deduce their evolutionary stages. Therefore, based on the comparison of ciliary patterns in strombidiids, their possible developing causes during evolution are presumed, and their evolutionary stages as well as evolutionary relevance are proposed.

In previous papers [12, 19, 21], the common ancestor of oligotrichs was hypothesized to have several longitudinal kineties which then were reduced to two kineties. These two kineties were assumed to be situated close to one another and shift to generate the pattern of *Parallelostrombidium* which was considered to be plesiomorphic relative to the pattern of kineties in other oligotrichs. However, this assumption does not agree with present and previous phylogenetic analyses based on SSrRNA gene sequences, in which species of *Parallelostrombidium* always branch late, while the subgenus *Novistrombidium* branches basally to other strombidiids with strong support [8, 25]. We agree with the general hypothesis that the somatic kineties in ancestral oligotrich were reduced to two kineties (the origin of GK and VK), but in our opinion, they should be separated rather than close to each other. Because in the ontogeny of strombidiids, the oral primordium is commonly situated between GK and VK, and the destinies of GK and VK are prominently different during the ontogenesis, i.e. GK spiraled with a dextral torsion of the oral primordium while VK remained longitudinally oriented, all this could have happened only when these two kineties were located separately in the ancestor. In addition, two separated kineties are also characteristic in *Lynnella semiglobulosa* [7] which branches basally to oligotrichs in phylogenetic trees (Fig 6), supporting that separated kineties are a plesiomorphy. Following our hypothesis, one of the two kineties which is near the proximal dextrally spiraled with a dextral torsion of the proximal end of the membranellar zone to form the GK, and the other located far away from the proximal remained longitudinally oriented on the ventral side to form the VK, as in the subgenus *Novistrombidium* (Fig 7). Based on the above analyses and molecular evidence (Fig 6), we suggest that the subgenus *Novistrombidium* pattern rather than the *Parallelostrombidium* pattern probably represents the ancestral state in the evolution of strombidiids. This hypothesis is supported by the ontogenetic pattern of known genera, in which the similar pattern of subgenus *Novistrombidium* is commonly recapitulated [12, 40]. By contrast, the closely parallel kineties in *Parallelostrombidium* are thus considered as a genus-specific feature that appears to have evolved convergently.

With further rightward torsion of the proximal in the ancestor, the anterior portion of the GK of the subgenus *Novistrombidium* pattern would extend to left and terminate at the left side of VK, giving rise to the pattern of the subgenus *Propecingulum* (Fig 7). This relative position of the anterior ends of GK and VK also appears in *Parallelostrombidium* and *Spirostrombidium*, and thus the *Propecingulum* pattern can be identified as probably ancestral to the patterns of the former genera. These were supported by the ontogenetic similarity in these three genera that the oral primordium is located between the stripes of extrusome attachment sites extending along anterior and posterior parts of girdle kinety respectively. The posterior GK of the *Propecingulum* pattern curved anteriad to lie parallel to the VK, giving rise to the type-a *Spirostrombidium* pattern (e.g. *S*. *cinctum*) as proposed in previous hypotheses, which then produced the type-b *Spirostrombidium* pattern (e.g. *S*. *schizostomum*) by extending the anterior end of GK to form two whorls (Fig 7). By contrast, the posterior GK of the *Propecingulum* pattern curved downward to lie parallel to the VK, giving rise to the pattern of the subgenus *Asymptokinetum*, which then produced the subgenus *Parallelostrombidium* pattern by orienting the anterior portion of VK obliquely to parallel with GK (Fig 7). In our phylogeny analyses, the close relationship of *Asymptokinetum* and *Propecingulum* is well confirmed, although *Spirostrombidium* is separated from *Propecingulum* (Fig 6).

We propose that the *Antestrombidium* pattern probably originated in parallel with *Omegastrombidium* pattern from the *Propecingulum* pattern (Fig 7). The strong evidence comes from the following. First, the circle kinety of *Antestrombidium* appears to be homologous to the Ω-shaped GK in *Omegastrombidium*. Second, the short extra kinety fragment usually located at the ventral center or around left shoulder area of cell in some individuals of *Omegastrombidium* (type-b pattern)[41] is generally similar to the GK of *Antestrombidium*. Furthermore, the orientation of the extra kinety matches that of the GK of *Antestrombidium* [41].

We hypothesize that the GK of *Antestrombidium* pattern and the extra kinety fragment of the *Omegastrombidium* type-b pattern, both of which have a dextrally spiraled orientation, were derived from the extended anterior portion of the GK of *Propecingulum*. This could have been accomplished by extension of the anterior portion of GK in *Propecingulum* followed by separation into two parts, the posterior part forming the Ω-shaped kinety by posterior migration of its right end and the anterior part developing into the GK of *Antestrombidium* and the extra kinety fragment of *Omegastrombidium* type-b. The *Omegastrombidium* type-a pattern that lacks an extra kinety fragment would have originated in an ancestor with the *Propecingulum* pattern in which the GK migrated directly without extending first. This hypothesis is well supported by our phylogenetic analyses, in which species of *Omegastrombidium* and *Antestrombidium* cluster together and species of *Propecingulum* branch basally (Fig 6). As in previous hypotheses [19], the *Omegastrombidium* type-a pattern gives rise successively to the *Apostrombidium* and *Varistrombidium* patterns (Fig 7). All genera derived from the *Propecingulum* pattern except for *Apostrombidium* cluster together in our phylogenetic analyses (Fig 6), which confirms their close evolutionary relationship; however, the trees do not reveal the relationship among them as yet.

*Strombidium*-like patterns constitute the other group in the evolutionary hypothesis of strombidiid oligotrichs (Fig 7). Three variations of the *Strombidium* pattern can be identified, characterized by the following different conformations of GK: 1) the type-a *Strombidium* pattern has a C-shaped GK with a small ventral gap, as in *S*. *capitatum*; 2) the type-b *Strombidium* pattern has a somatic ciliature with a closed GK that is typical for *Strombidium*, as in *S*. *sulcatum*; and 3) the Type-c *Strombidium* pattern has a ventral open GK, with its right end curved slightly toward the posterior, as in *S*. *apolatum*. We agree with Agatha’s [19] hypothesis that the type-a *Strombidium* pattern was derived first from *Novistrombidium* by migration of the left portion of GK toward the anterior. Three further evolutionary lineages (Fig 7) were suggested by our analyses. First, the C-shaped GK of the type-a *Strombidium* pattern closed, forming the circular GK of the type-b *Strombidium* pattern. *Foissneridium* and *Opisthostrombidium* then developed from the type-b *Strombidium* pattern as suggested by Agatha [19] when the horizontal GK migrated toward the posterior below the oral primordium, with the stripe of extrusome attachment sites above or below the oral primordium. Second, the *Williophrya* pattern originated when the GK of the type-a *Strombidium* pattern split dorsally into two fragments, together with loss of the VK. This hypothesis is corroborated by our molecular analyses, in which *Williophrya* nested within *Strombidium* (Fig 6). Third, the right end of GK in an ancestor with the type-a *Strombidium* pattern curved toward the posterior, producing the type-c *Strombidium* pattern. The *Sinistrostrombidium* pattern was assumed to originate from the *Strombidium* pattern by elongation and sinistral spiraling of the right portion of GK. This could be confirmed by the transition stage type-c *Strombidium* patterns. In our phylogenetic trees, the association of *Sinistrostrombidium* with *Strombidium subtropicum* which has a type-c pattern [42], strongly supports this hypothesis (Fig 6).

In general, our evolutionary hypothesis of ciliary patterns in strombidiids is supported by the molecular phylogeny. The two hypothetical evolutionary pathways are in basic agreement with the topology of the two main strombidiid clades; however, some evolutionary relationships within these clades (e.g., *Apostrombidium* and *Omegastrombidium* patterns are closely related in the hypothesis) are not supported by the molecular tree, and the nesting of tontoniids within strombidiid clade II (Fig 6) does not match the morphological analysis. Previous evolutionary hypotheses also were tested by molecular phylogenetic analyses [19, 21], which yielded the same poor match between morphological and molecular evidence. At present, topologies of trees constructed from available sequences of oligotrichs tend to vary, depending on number of samples included or the analytic methods used. This, as well as the low support values for basal nodes in all molecular studies performed so far, suggests that there are still many gaps in the molecular evidence for SSrRNA trees to provide an appropriately rigorous test of conclusions inferred from morphological inference.

Beside the morphological and molecular evidence, some ontogenetic data supply important support for the evolutionary hypothesis in our study. However, the detailed ontogeny of most species are not available at present. With the increase of more comprehensive data combining morphology, molecular, ontogeny and ultrastructure, we can better understand about the evolution of the girdle kinty patterns in strombidiids.

### Remarks on the Phylogenetic Position of *Lynnella*

The family Lynnellidae was established for the morphospecies *Lynnella semiglobulosa* [7], but its suprafamilial classification has remained uncertain. *Lynnella* has an open AZM like that of oligotrichs but lacks the differentiation of AM and VM, which is characteristic of them [7]. Furthermore, its ellipsoidal macronuclear nodules and longitudinally arranged somatic kineties are characteristic features of choreotrichs.

**Fig 8:**
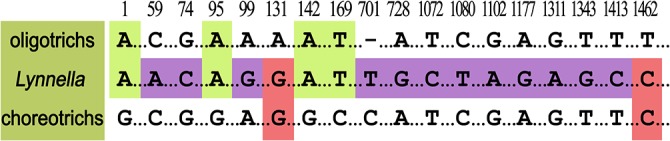
Unique nucleotide signatures in the SSrRNA gene sequences of oligotrichs, *Lynnella*, and choreotrichs. Positions of nucleotides in the alignment are given at the top. Gaps are indicated by dashes.

In previous studies, the position of *Lynnella* has not been stable in phylogenetic trees. It clustered with oligotrichs in some analyses [8, 24] and with choreotrichs in others [7, 9, 21, 39], albeit with relatively higher support in ML and BI trees (95/0.91 and 79/1.00) for the former relationship vs. 65/0.61, 79/1.0 and 71/0.99 for the latter. In the present study, *Lynnella* clusters with oligotrichs in a basal position in the ML tree with low support, but in BI trees, the relationship between *Lynnella*, oligotrichs, and choreotrichs is completely unresolved. In addition, our analyses showed that neither hypothesis of *Lynnella* falling into oligotrich clade nor choreotrich clade was rejected by AU (*P =* 0.169 and 0.086, respectively) or SH tests (*P =* 0.742 and 0.438, respectively). In summary, therefore, it remains impossible to assign *Lynnella* to a subclass. Analysis of molecular characters from the SSrRNA alignment reveals four unique nucleotide identities between *Lynnella* and oligotrichs, and two between *Lynnella* and choreotrichs (Fig 8). Moreover, 12 nucleotides in the sequence of *Lynnella* differ from both oligotrichs and choreotrichs (Fig 8). This evidence suggests that *Lynnella* may represent an evolutionary lineage separate from both the subclasses Oligotrichia and Choreotrichia, but more data will be needed to confirm this.

In regard to its ordinal classification, *Lynnella* can be excluded from all three known orders in oligotrichs s. lat. by the following differences: 1) Oligotrichida has an adoral zone of membranelles with differentiation of anterior and ventral membranelles, but ventral membranelles are absent in *Lynnella*; 2) in Choreotrichida, the adoral zone of membranelles form a closed circle, but they are open on the ventral side in *Lynnella*; and 3) Tintinnida has a lorica covering the cell body, but there is no lorica in *Lynnella*) [23]. Furthermore, *Lynnella* does not cluster reliably with any of the known orders of oligotrichs s. lat. in any phylogenetic analysis based on SSrRNA gene sequences [7–9, 21, 25]. Therefore, there is more than enough evidence to justify establishment of a new order, Lynnellida ord. nov. for *Lynnella*. In a previous paper [11], we classified *L*. *semiglobulosa* into the order Lynnellida but did not provide a formal description of the new order. This omission is corrected here.

Class Spirotrichea

Order Lynnellida ord. nov.

Diagnosis. Adoral zone of membranelles open without differentiation of anterior and ventral membranelles.

Type family. Lynnellidae Liu *et al*., 2011.

Remarks. In choreotrichs, a minute ventral gap like that in *Lynnella* also appears in the AZM of *Parastrombidium* and *Parastrombidinopsis*, and in trees based on morphological characters alone [9], *Lynnella* is an unresolved sister to both genera in a moderately supported clade. These three genera thus were considered to have a close phylogenetic relationship, and the ventrally opened AZM was interpreted as synapomorphic retrogression to the plesiomorphic (open) state [9, 21]. However, there are obvious differences between *Lynnella*, *Parastrombidium* and *Parastrombidinopsis* in the AZM. In *Lynnella*, the proximal end of the AZM is located in the oral cavity below the distal end, and its endoral membrane is positioned longitudinally on the inner wall of the buccal lip, which is more like its appearance in oligotrichs and probably represents a plesiomorphy. By contrast, the AZMs of *Parastrombidium* and *Parastrombidinopsis* are only slightly opened, with the proximal end on the same horizontal level as the distal end, and the endoral membrane is absent or extends across the peristomial field, which is characteristic of other choreotrichs and considered to be a secondary apomorphy. These differences suggest that *Lynnella* is not closely related to *Parastrombidium* and *Parastrombidinopsis*, and may represent a divergent, plesiomorphic taxon either within the Oligotrichia or independent of both oligotrichs and choreotrichs. In our phylogenetic analyses, *Lynnella* is separated from *Parastrombidinopsis* (Fig 6), which indicates a deep divergence between them.

## References

[1] Dale T, Dahl E. Mass occurrence of planktonic oligotrichous ciliates in a bay in southern Norway. J Plankton Res. 1987;9:871–879.

[2] Pierce RW, Turner JT. Ecology of planktonic ciliates in marine food webs. Rev Aquat Sci. 1992;6:139–181.

[3] Fenchel T. The microbial loop– 25 years later. J Exp Mar Biol Ecol. 2008;366:99–103.

[4] Liu W, Xu D, Lin X, Li J, Gong J, Al-Rasheid KA, et al *Novistrombidium sinicum* n. sp. and *Novistrombidium orientale* n. sp. (Protozoa: Ciliophora): Two new oligotrich ciliates from a mangrove wetland, South China. J Eukaryot Microbiol. 2009;56:459–465. 10.1111/j.1550-7408.2009.00425.x 19737199

[5] McManus GB, Xu D, Costas BA, Katz LA. Genetic identities of cryptic species in the *Strombidium stylifer*/*apolatum*/*oculatum* cluster, including a description of *Strombidium rassoulzadegani* n. sp. J Eukaryot Microbiol. 2010;57:369–378. 10.1111/j.1550-7408.2010.00485.x 20553354

[6] Agatha S. Global diversity of aloricate Oligotrichea (Protista, Ciliophora, Spirotricha) in marine and brackish sea water. PloS ONE. 2011;6:e22466 10.1371/journal.pone.0022466 21853034 PMC3154192

[7] Liu W, Yi Z, Lin X, Al-Rasheid KA. Morphologic and molecular data suggest that *Lynnella semiglobulosa* n. g., n. sp. represents a new family within the subclass Choreotrichia (Ciliophora, Spirotrichea). J Eukaryot Microbiol. 2011;58:43–49. 10.1111/j.1550-7408.2010.00519.x 21129086

[8] Liu W, Yi Z, Warren A, Al-Rasheid KAS, Al-Farraj SA, Lin X, et al Taxonomy, morphology and molecular systematics of a new oligotrich ciliate, *Williophrya maedai* gen. nov., sp. nov., with redescriptions of *Strombidium basimorphum* and *Pseudotontonia simplicidens* (Protozoa, Ciliophora, Oligotrichia). Syst Biodiver. 2011;9:247–258.

[9] Agatha S, Struder-Kypke MC. Reconciling cladistic and genetic analyses in choreotrichid ciliates (ciliophora, spirotricha, oligotrichea). J Eukaryot Microbiol. 2012;59:325–350. 10.1111/j.1550-7408.2012.00623.x 22646795

[10] Xu D, Sun P, Shin MK, Kim YO. Species boundaries in tintinnid ciliates: a case study—morphometric variability, molecular characterization, and temporal distribution of *Helicostomella* species (Ciliophora, Tintinnina). J Eukaryot Microbiol. 2012;59:351–358. 10.1111/j.1550-7408.2012.00625.x 22591516

[11] Song W, Li J, Liu W, Jiang J, Al-Rasheid KA, Hu X. Taxonomy, morphology and molecular systematics of three oligotrich ciliates, including a description of *Apostrombidium parakielum* spec. nov. (Ciliophora, Oligotrichia). Inter J Syst Evol Microbiol. 2013;63:1179–1191. 10.1099/ijs.0.048314-0 23291887

[12] Agatha S. Evolution of ciliary patterns in the Oligotrichida (Ciliophora, Spirotricha) and its taxonomic implications. Zoology. 2004;107:153–168. 16351935 10.1016/j.zool.2004.02.003 PMC2848327

[13] Lynn D. The Ciliated Protozoa. Characterization, Classification, and Guide to the Literature. New York: Springer 2008. 605 p.

[14] Claparède E, Lachmann J. Études sur les Infusoires et les Rhizopodes. Mém Inst Nat Genevois. 1859;6:261–482.

[15] Petz W, Song W, Wilbert N. Taxonomy and ecology of the ciliate fauna (Protozoa, Ciliophora) in the endopagial and pelagial of the Weddell Sea, Antarctica. Stapfia. 1995;40:1–223.

[16] Song W, Bradbury PC. Studies on some new and rare reported marine planktonic ciliates (Ciliophora: Oligotrichia) from coastal waters in North China. J Mar Biol Ass UK. 1998;78:767–794.

[17] Agatha S. Morphology and ontogenesis of *Novistrombidium apsheronicum* nov. comb. and *Strombidium arenicola* (Protozoa, Ciliophora): a comparative light microscopical and SEM study. Europ J Protistol. 2003;39:245–266.

[18] Song W, Warren A, Hu X. Free-living ciliates in the Bohai and Yellow Seas Beijing: Science Press 2009. 518 p.

[19] Agatha S. Updated hypothesis on the evolution of oligotrichid ciliates (Ciliophora, Spirotricha, Oligotrichida) based on somatic ciliary patterns and ontogenetic data. Europ J Protistol. 2011;47:51–56. 10.1016/j.ejop.2010.09.001 PMC3034029 20961741

[20] Xu D, Sun P, Clamp J, Ma H, Song W. The establishment of a new oligotrich genus *Varistrombidium* gen. nov. and the morphology and phylogeny of a marine ciliate, *Varistrombidium kielum* (Maeda and Carey, 1985) nov. comb. (Protista, Ciliophora). Acta Zootaxon Sin. 2011;36:502–511.

[21] Agatha S, Struder-Kypke M. What morphology and molecules tell us about the evolution of Oligotrichea (Alveolata, Ciliophora). Acta Protozool. 2014;53:77–90.

[22] Wilbert N. Eine verbesserte Technik der Protargolimprägnation für Ciliaten. Mikrokosmos. 1975;64:171–179.

[23] Agatha S. A Cladistic Approach for the classification of oligotrichid ciliates (Ciliophora: Spirotricha). Acta Protozool. 2004;43:201–217. 20396404 PMC2854820

[24] Xu D, Sun P, Warren A, Noh JH, Choi DL, Shin MK, et al Phylogenetic investigations on ten genera of tintinnid ciliates (Ciliophora: Spirotrichea: Tintinnida), based on small subunit ribosomal RNA gene sequences. J Eukaryot Microbiol. 2013;60:192–202. 10.1111/jeu.12023 23346918

[25] Liu W, Yi Z, Li J, Warren A, Al-Farraj SA, Lin X. Taxonomy, morphology and phylogeny of three new oligotrich ciliates (Protozoa, Ciliophora, Oligotrichia) from southern China. Inter J Syst Evol Microbiol. 2013;63:4805–4817. 10.1099/ijs.0.052878-0 24096352

[26] Medlin L, Elwood HJ, Stickel S, Sogin ML. The characterization of enzymatically amplified eukaryotic 16S-like rRNA-coding regions. Gene. 1988;71:491–499. 3224833 10.1016/0378-1119(88)90066-2

[27] Jeanmougin F, Thompson J, Gouy M, Higgins D, Gibson T. Multiple sequence alignment with Clustal X. Trends Biochem Sci. 1998;23:403–405. 9810230 10.1016/s0968-0004(98)01285-7

[28] Hall TA. BioEdit: a user-friendly biological sequence alignment editor and analysis program for Windows 95/98/NT. Nucleic Acids Symp Ser. 1999;41:95–98.

[29] Nylander JA. MrModeltest Ver.2. Evolutionary Biology Centre, Uppsala University, Sweden 2004.

[30] Posada D, Crandall KA. Modeltest: testing the model of DNA substitution. Bioinformatics. 1998;14:817–818. 9918953 10.1093/bioinformatics/14.9.817

[31] Ronquist F, Huelsenbeck JP. MrBayes 3: Bayesian phylogenetic inference under mixed models. Bioinformatics. 2003;19:1572–1574. 12912839 10.1093/bioinformatics/btg180

[32] Guindon S, Gascuel O. A simple, fast and accurate algorithm to estimate large phylogenies by maximum likelihood. Syst Biol. 2003;52:696–704. 14530136 10.1080/10635150390235520

[33] Swofford DL. PAUP*. Phylogenetic analysis using parsimony (*and other methods). Version 4. Sunderland, MA. 2002.

[34] Page RDM. TREEVIEW: an application to view phylogenetic trees on personal computers. Comput Appl Biosci. 1996;12:357–358. 8902363 10.1093/bioinformatics/12.4.357

[35] Tamura K, Dudley J, Nei M, Kumar S. MEGA4: molecular evolutionary genetics analysis (MEGA) software version 4.0. Mol Biol Evol. 2007;24:1596–1599. 17488738 10.1093/molbev/msm092

[36] Shimodaira H. An approximately unbiased test of phylogenetic tree selection. Syst Biol. 2002;51:492–508. 12079646 10.1080/10635150290069913

[37] Shimodaira H, Hasegawa M. CONSEL: for assessing the confidence of phylogenetic tree selection. Bioinformatics. 2001;17:1246–1247. 11751242 10.1093/bioinformatics/17.12.1246

[38] Gao S, Gong J, Lynn D, Lin X, Song W. An updated phylogeny of oligotrich and choreotrich ciliates (Protozoa, Ciliophora, Spirotrichea) with representative taxa collected from Chinese coastal waters. Syst Biodiver. 2009;7: 235–242.

[39] Li J, Liu W, Gao S, Warren A, Song W. Multigene-based analyses of the phylogenetic evolution of oligotrich ciliates, with consideration of the internal transcribed spacer 2 secondary structure of three systematically ambiguous genera. Eukaryot Cell. 2013;12: 430–437. 10.1128/EC.00270-12 23314963 PMC3629766

[40] Xu D, Song W, Warren A. Morphology and infraciliature of two new species of marine oligotrich ciliates (Ciliophora: Oligotrichida) from China. J Nat Hist. 2006;40:1287–1299.

[41] Song W, Wang M, Warren A. Redescriptions of three marine ciliates, *Strombidium elegans* Forentin,1901, *Strombidium sulcatum* Claparede & Lachmann,1859 and *Heterostrombidium paracalkinisi* Lei, Xu & Song, 1999 (Ciliophora, Oligotrichida). Europ J Protistol. 2000;36:327–342.

[42] Liu W, Yi Z, Lin X, Li J, Al-Farraj AS, Al-Rasheid KA, et al. Morphology and molecular phylogeny of three new oligotrich ciliates (Protozoa, Ciliophora) from the South China Sea. Zool J Linn Soc. 2015;Accepted.

